# Impact of prenatal maternal dietary exclusion on childhood obesity and overweight risk

**DOI:** 10.1371/journal.pone.0297614

**Published:** 2024-03-06

**Authors:** Brenton Horne, Enamul Kabir, Khorshed Alam

**Affiliations:** 1 School of Mathematics, Physics and Computing, University of Southern Queensland, Toowoomba, Queensland, Australia; 2 School of Business and Centre for Health Research, University of Southern Queensland, Toowoomba, Queensland, Australia; Texas A&M University College Station, UNITED STATES

## Abstract

**Background:**

Child birthweight is a measure of fetal nutrition that is primarily determined by prenatal maternal (PM) diet. Child birthweight and child obesity/overweight risk are well established to be linked. Nevertheless, no studies have investigated the impact of PM dietary exclusion on child obesity/overweight risk or body mass index z-score (BMIz).

**Objectives:**

The study aimed to determine whether PM dietary exclusion affected the child’s BMIz, obesity/overweight risk, whether child birthweight serves as a mediator of this, and whether PM use of dietary supplements can protect against this.

**Methods:**

Waves within the years 2004–2019 from the Longitudinal Study of Australian Children, a population-based cohort study, were analyzed. The participants were aged 0 to 15 years during these waves of the study. Analysis was conducted using logistic and linear models. A total of 5,107 participants were involved in the first wave of the study.

**Results:**

The PM exclusion of fish was associated with a higher risk of being underweight at age 14 or 15 years and mild-to-moderate obesity at age 6 or 7 years. The PM exclusion of egg was associated with a higher risk of being overweight at age 14 or 15 years. The exclusion of dairy was associated with more mixed effects. Mediation effects did not reach statistical significance. Moderation effects involving PM dietary supplement use, when they did occur, were associated with higher child BMIz and usually a higher risk of obesity/overweight.

**Conclusions:**

Fish and eggs are likely important parts of PM diets for preventing childhood obesity and overweight. Further studies will be needed to determine reasons for this and the apparent adverse effects of dietary supplements on overweight/obesity risk.

## Introduction

Childhood obesity and overweight are a major global health crisis in the 21^st^ Century. The conditions contribute to numerous causes of morbidity and mortality, including cardiovascular disease, type 2 diabetes mellitus, and certain cancers [1]. It is particularly prevalent in Australia, where 24% of children were overweight or obese between 2017 and 2018 [2]. Thus, attempting to combat these forms of malnutrition is essential for the wellbeing of society. An important part of such attempts is identifying modifiable risk factors for the condition, such as the dietary habits of mothers during pregnancy. The mechanisms by which these risk factors achieve their effects also need to be identified because these mechanisms can pave additional research avenues for reducing the risk of childhood obesity and overweight. Protective factors have been investigated for this same reason.

A well-established relationship exists between fetal malnutrition and obesity and overweight risk later in life [3, 4]. For instance, young men exposed to famine during the first half of their mothers’ pregnancy, as during the Dutch famine of 1944–1945, were found to have a higher obesity risk [5]. However, the opposite effect was observed when these men were exposed to starvation during the last half of pregnancy or infancy [5]. Child birthweight appears to mediate the effect of fetal malnutrition on subsequent obesity and overweight risk [3, 4]. According to the Developmental Origins of Health and Disease (DOHaD) hypothesis, this is explained by prenatal malnutrition adapting the child’s metabolism to cope with a similar postnatal environment [6]. A mismatch between the prenatal and postnatal environment can lead to conditions such as childhood obesity and overweight [6]. Studies on animals have found evidence of an effect of prenatal maternal (PM) diet on postnatal metabolic outcomes in offspring [7]. There have been studies that have investigated the effects of PM use of dietary supplements on child obesity and overweight [8]. For instance, one study in Nepal has shown that folate, iron, and zinc supplementation during pregnancy is associated with reduced skinfold thickness (a measure of adiposity) in children aged 6–8 years [9]. This finding suggests that taking these supplements during pregnancy may have a protective effect against childhood obesity.

Meat and fish are rich sources of iron and zinc [10, 11], so the aforementioned Nepalese study provides a rationale for suspecting that the exclusion of these foods may be associated with an increased risk of childhood obesity and overweight. Dairy, meat, fish, and eggs are all sources of vitamin B12 [12]. Dairy, eggs, and liver are also rich sources of choline [13]. The one-carbon cycle, which provides methyl groups for deoxyribonucleic acid (DNA) methylation, involves vitamin B12 and choline, among other nutrients (such as folate), and has been implicated in the mechanisms of DOHaD. Alterations in this cycle can lead to epigenetic changes that permanently program the fetus’ organs to be ready for a postnatal environment of nutritional deprivation [14]. Nevertheless, to the best of the authors’ knowledge, no previous study on humans has specifically examined the effect of PM dietary exclusion (PMDE) on childhood obesity and overweight risk or body mass index z-score (BMIz). Accordingly, the present study aimed to address this notable research gap. The data used were obtained from a longitudinal study conducted in Australia, where children under the age of two years were followed since 2004. The primary caregivers (usually the mother) were interviewed to collect various information regarding the mother’s pregnancy, specifically during wave 1 in 2004.

This study investigated the effect of excluding certain food items from the mother’s diet during pregnancy on the risk of children later developing childhood obesity and overweight and on their BMIz. Child birthweight was investigated as a possible mediating factor in this relationship. PM dietary supplement use was examined as a possible protective factor against the effects of excluding food items from the mother’s prenatal diet on childhood obesity and overweight risk.

## Materials and methods

### Ethics approval

This study (consisting entirely of secondary data analysis) received ethics approval from the University of Southern Queensland’s Human Research Ethics Committee with the project ID ETH2023-0175.

### Data

Data were obtained from the Longitudinal Study of Australian Children (LSAC). The design of the LSAC is described elsewhere [15]. Briefly, LSAC started in 2004 and is an ongoing two-yearly prospective cohort study. It had two cohorts, namely, B (for “baby”) and K (for “kindergarten”). Cohort B focused on children aged <2 years in 2004.Cohort K focused on children aged 4 or 5 years in 2004. Only cohort B was analyzed in this study because it had the required PM details. In 2004, the first wave of LSAC was conducted. The second wave was conducted in 2006, the third wave in 2008, and so forth. Two-stage cluster sampling was used to obtain the sample that LSAC used. LSAC received ethics approval from the Australian Institute of Family Studies Ethics Committee and written parental consent was obtained for all participants.

The majority of the data analyzed in this study were collected by trained interviewers through in-person interviews with the child’s primary caregiver. The child’s weight was measured to the nearest 50 g using glass bathroom scales, and the child was wearing light clothing. In waves 2 and 3, the scales used were Salter Australia glass bathroom scales (150 kg × 50 g) and HoMedics digital body mass index (BMI) bathroom scales (180 kg × 100 g) [16 p8]. For waves 4 to 8, Tanita body fat scales were used [16 p8]. The child’s height was measured to the nearest 0.1 cm by using a portable rigid stadiometer. In waves 2 and 3, an Invicta stadiometer from Modern Teaching Aids was used to find the child’s height [16 p8]. In waves 4 to 8, a laser stadiometer was used to measure the child’s height [16 p8]. Two height measurements were taken, and if they differed by 0.5 cm or more, a third measurement was taken. The two closest measurements were then averaged and included in the dataset as the child’s height [16 p8]. These measurements were used to calculate the child’s BMI.

Data contained within the LSAC restricted data set were accessed from the Australian Data Archives on 21 March 2023 [17]. The data set accessed was de-identified by its owner, the Australian Government Department of Social Services. However, the data contained sufficient information to potentially re-identify participants.

### Variables

BMIz and weight status served as outcome variables. Dietary exclusion variables were treated as risk factor variables. Dietary supplement variables were treated as moderator variables, and child birthweight was treated as a mediator variable. These risk factors, mediator, and moderator variables were all recorded in wave 1 only. The outcome variables were recorded through waves 2–8. Information on variables used in the analysis, including covariates, is provided in Table 1.

**Table 1:** Variables used in study.

Variable	Wave(s)	Type	Description and/or justification
**Covariates**			
Child sex	1	DC^a^	Controlled due to sex differences in childhood obesity/overweight risk [18].
PM^b^ DM^c^			1 meant yes; 2 meant no. Gestational diabetes is a known risk factor for childhood obesity [19].
PM hypertension			Same coding as PM DM. PM hypertension is a known risk factor for childhood obesity [19, 20].
PM depression/anxiety/stress			Same coding as PM DM. PM stress is a known risk factor for childhood obesity [21].
PM antibiotic use			Same coding as PM DM. Controlled for as a proxy for PM infection [22].
Child ate breakfast	2–8^d^		Only pertains to the day of interview. 0 means yes and 1 means no. The primary caregiver gave the answer for waves 1–5; for waves 6–8 the child provided this information. Controlled for due to evidence that skipping breakfast is associated with higher childhood obesity and overweight risk [23].
Child has sleep issues	1–8		0 for yes and 1 for no. Controlled for due to evidence for the role of sleep in childhood obesity and overweight risk [24].
Maternal age at child’s birth	1	Q^e^	Calculated from date of birth of mother and child. Was correlated with BMIz in some studies [25].
Maternal BMI at wave 1			Closest recorded variable to pre-pregnancy BMI, which is known to be correlated with childhood obesity and overweight risk [25].
Breastfeeding cessation age	1–3		In units of days. If the child is still breastfeeding, their current age is used. Controlled for as breastfeeding is protective against childhood obesity and overweight [26].
Parental hostility version 3	2–3		Average of the parent has (each pertaining to the last 6 months and rated from 1 to 10 in ascending order of frequency): been angry with the child; lost their temper with the child; and shouted at the child. Controlled for due to the role of parenting in childhood obesity/overweight risk [27].
Days/week ≥30 mins exercise	7–8		Pertains to the child; information provided by child. Only physical activity of at least moderate intensity is included in this. Controlled for due to obesity/overweight being fundamentally an energy imbalance issue.
Days/week ≥60 mins exercise			
Child alcohol consumption			Number of drinks the child has had in the last week as reported by the child. Controlled for due to some evidence of a correlation with child obesity/overweight risk [28].
Parental warmth	1–8		Average of how often the parent has (each pertaining to the last 6 months and rated from 1 to 5 in ascending order of frequency): told the child how happy they make them; held/hugged the child for no specific reason; had close moments with the child; enjoyed listening/doing things with the child; expressed physical affection for the child; and felt close to the child when the child was upset. Some studies have found an effect of parenting style on child BMI [27].
Child age			Age disparities in childhood obesity/overweight risk [29].
Parental weekly income			Part of socioeconomic status (SES), which is known to be associated with childhood obesity/overweight [30].
Parental highest qualification		MC^f^	Part of SES. Coded as: 1 for postgraduate degree; 2 for graduate diploma/certificate; 3 for bachelor’s degree; 4 for (advanced) diploma; 5 for trade certificate; and 6 for others.
Parental highest schooling			Part of SES. Coded as: 1 for year 12 or equivalent; 2 for year 11 or equivalent; 3 for year 10 or equivalent; 4 for year 9 or equivalent; 5 for year ≤8; 6 for never attended school; and 7 for still at school.
Parental occupation			4-digit Australia and New Zealand Standard Classification of Occupations (ANZSCO) code. Part of SES.
Cigarettes mom smoked/day during each pregnancy trimester	1		Coded as: 0 for never; 1 for ≤10; 2 for 11–20; 3 for 21–30; 4 for 31–40; 5 for 41–50; 6 ≥51; and 9 for occasional, not every day. PM smoking is a risk factor for childhood obesity/overweight [31].
Parental smoking frequency	1–3, 5–8		Coded as: 1 for does not smoke at all; 2 for <1/day; and 3 for ≥1/day. Parental smoking has been associated with an increased risk of childhood obesity and overweight in some studies [32].
Child ate fresh fruit	2–8		Only pertains to 24 hours before the interview and is the number of times they have consumed the food/drink in question. Controlled for due to the role of diet in childhood obesity and overweight. 0 means none; 1 means once; 2 means twice; and 3 means thrice or more. These last two categories are merged into a single category coded as 2 for wave 2. Controlled for as obesity and overweight is known to be fundamentally an energy imbalance [33].
Child drank fresh juice			
Child ate raw vegetables			
Child ate cooked vegetables			
Child ate processed meat			
Child ate hot chips			
Child ate snack food			
Child ate sugary food			
Child ate full milk products			
Child ate skim milk products			
Child drank water			
Child drank SSBs^g^			
How child spends spare time	2–7		Categorized as: 1 for inactive pastimes; 2 for inactive/active pastimes equally likely; and 3 for active pastimes. Obesity and overweight is fundamentally an energy imbalance, hence why this is controlled for [33].
Child enjoys physical activity	3–5		Rated from 1 to 5; higher the value the more the child enjoys physical activity.
How often TV is on during meals	2.5, 3.5, 5–8		2.5/3.5 refers to data from between wave questionnaires that were sent out to parents. For these waves the variables recorded took on values of 1 (for always) to 5 (for never). A similar scale was used for the remaining waves, except with the order reversed. Between wave variables were used in the analysis of the wave that came immediately after. Known risk factor for childhood obesity and overweight [34].
Child sleep duration adequacy	6–8		Pertains to the last month and was reported by child. Coded from 1 to 5 in descending order of adequacy. Controlled for due to some studies that showed a relationship between sleep parameters and child obesity and overweight [35].
Child sleep quality			Same as for duration adequacy, except ranked in descending order of quality.
**Independent variables**			
PM dietary exclusion of meat	1	DC	Each of these variables are individually coded as: 0 for no; 1 for yes.
PM dietary exclusion of fish			
PM dietary exclusion of dairy			
PM dietary exclusion of eggs			
PM dietary exclusion of other foods			
**Mediator variable**			
Child birthweight	1	Q	Controlled for due to evidence of a correlation with child obesity risk [36–38].
**Moderator variables**			
PM Rx^h^ iron supplement use	1	DC	Each of these variables are individually coded as: 0 for no; 1 for yes. For instance, if prescription iron supplements were used the PM Rx iron supplement use variable will be recorded as 1.
PM OTC^i^ iron supplement use			
PM OTC folate supplement use			
PM other dietary supplement use			
**Outcome and related variables**			
BMI^j^ z-score (BMIz)	2–8	Q	Based on US Centers for Disease Control and Prevention (CDC) and UK 1990 growth reference data [39].
BMI percentile (BMIpct)			CDC growth reference data were used to calculate these percentiles.
Weight status		MC	BMIpct<5 was classed as underweight and coded as 0; 5 ≤ BMIpct < 85 was classed as healthy weight and coded as 1; 85 ≤ BMIpct < 95 was classed as overweight and coded as 2; 95 ≤ BMIpct < 99 was classed as mild-to-moderate obesity and coded as 3; 99 ≤ BMIpct was classed as severe obesity and coded as 4 [33].
Binary weight status		DC	Calculated based on weight status. 0 corresponds to healthy weight and 1 corresponds to overweight or obesity. Underweight (defined as those with a BMI percentile of less than 5) children were excluded from the analyses involving binary weight status.

DC: dichotomous.

PM: prenatal maternal.

DM: diabetes mellitus.

This refers to waves 2 to 8. In other words, the child ate breakfast variable was recorded for waves 2, 3, 4, 5, 6, 7 and 8.

Q: quantitative.

MC: multicategorical.

SSBs: sugar-sweetened beverages.

Rx: prescription.

OTC: over the counter.

BMI: body mass index.

**Fig 1:**
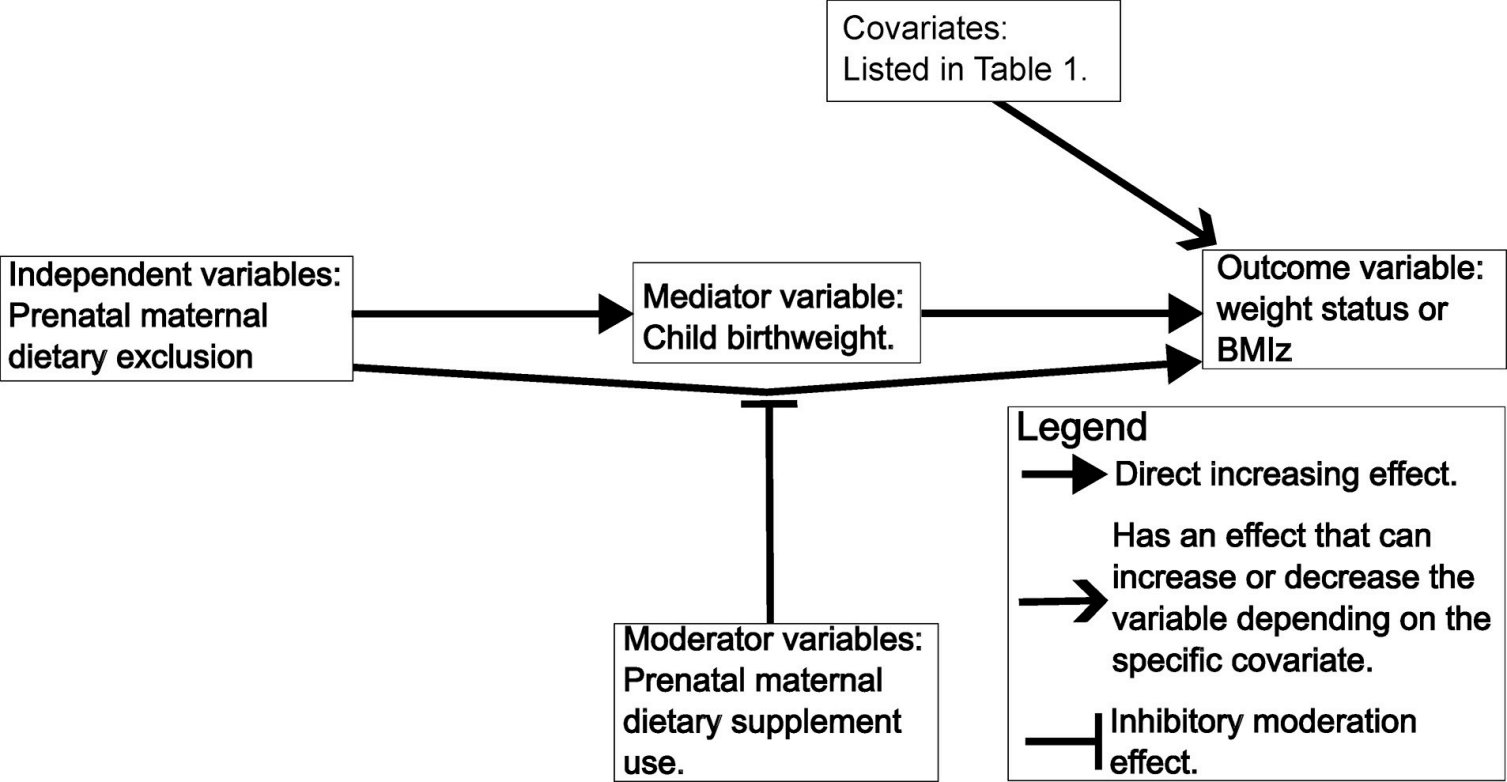
Proposed relationship between variables.

Fig 1 is a flowchart describing the examined relationship between the variables. The independent variables pertaining to the PMDE were hypothesized to affect the outcome variables of weight status and BMIz, at least partially, through the mediator variable child birthweight. The moderator variables pertaining to PM dietary supplement use were hypothesized to reduce the effect of the independent variables on the outcome variables. The covariates were expected, based on previous research, to potentially affect the outcome variables too, so they must be controlled for.

### Data cleaning

Children with missing data in the variables analyzed were excluded from the analysis. Children that had these variables recorded with nonsensical negative values that were not explained in the data dictionary were also excluded from the analysis. Multiple imputation was attempted using the mice R package as a means of filling in the missing data, but singular matrix errors prevented its use. The misty R package was used to conduct Little’s Missing Completely at Random test [40]; however, the results were inconclusive due to singular matrix errors.

### Statistical analysis

A p-value of < 0.05 served as the cutoff for statistical significance. Two-sided significance testing was used throughout the analysis. Regression models fitted were all fixed effects, univariate and multivariable. R version 4.3.0 and 4.3.1 were used to perform all the analyses.

To each wave for which the outcome variables were recorded, linear models (LMs) were fitted to test whether the hypothesized risk factors were correlated with child BMIz. Similarly, to each wave, multinomial logistic models (MLMs) were fitted to test whether the hypothesized risk factors were associated with weight status. The VGAM library was used to fit the MLMs. These models were used to determine whether any PMDE variables were correlated with child BMIz or overweight or obesity risk.

The LMs included as regressors the PMDE variables and all the covariates listed in Table 1. The MLMs included as regressors the PMDE variables, current parental BMI, wave 1 maternal BMI, and child age.

Univariate moderation analysis was conducted using version 4.3 of the PROCESS R macro developed by Andrew F. Hayes [41] with the outcome variables of BMIz and binary weight status. When binary weight status was the outcome variable, underweight children were excluded from the analysis because this allowed the two categories used in the analysis to be overweight/obese and healthy weight. Univariate mediation analysis was conducted using the method described in Iacobucci (2012) [42] with outcome variables of BMIz and weight status.

## Results

### Participant demographics

Table 2 summarizes the demographics of the participants before and after cases with missing data removed. Indigenous Australian children were found to be under-represented in the sample across all waves after excluding cases with missing data, suggesting that the data were likely not missing completely at random. Table 3 summarizes the missing values for variables of interest.

**Table 2:** Demographics of participants included in analysis before/after excluding subjects with missing data.

Characteristic \ Wave	1	2	3	4	5	6	7	8
Number	5,107 / 3,887	4,606 / 1,008	4,386 / 918	4,242 / 953	4,085 / 1,145	3,764 / 1,073	3,381 / 798	3,127 / 891
Age in years, median (IQR)	0.769 (0.296) / 0.769 (0.290)	2.85 (0.326) / 2.84 (0.315)	4.84 (0.325) / 4.83 (0.285)	6.84 (0.416) / 6.84 (0.389)	8.92 (0.422) / 8.90 (0.397)	10.9 (0.485) / 10.9 (0.498)	13.0 (0.518) / 12.9 (0.470)	14.8 (0.456) / 14.8 (0.444)
Indigenous children, no (%)	230 (4.50) / 126 (3.24)	180 (3.90) / 5 (0.496)	149 (3.40) / 6 (0.654)	145 (3.42) / 10 (1.05)	139 (3.40) / 12 (1.05)	106 (2.82) / 9 (0.839)	87 (2.57) / 5 (0.627)	79 (2.53) / 8 (0.898)
Male sex, no (%)	2,608 (51.1) / 2,010 (51.7)	2,349 (51.0) / 512 (50.79)	2,251 (51.3) / 463 (50.4)	2,187 (51.6) / 499 (52.4)	2,096 (51.3) / 558 (48.7)	1,929 (51.2) / 531 (49.5)	1,734 (51.3) / 401 (50.3)	1,606 (51.4) / 452 (50.7)
Child born in Australia, no (%)	5,088 (99.6) / 3,874 (99.7)	4,589 (99.6) / 1,005 (99.7)	4,370 (99.6) / 915 (99.7)	4,227 (99.6) / 949 (99.6)	4,070 (99.6) / 1,140 (99.6)	3,749 (99.6) / 1,071 (99.8)	3,371 (99.7) / 796 (99.7)	3,117 (99.7) / 888 (99.7)
Mom born in Australia, no (%)	3,989 (78.1) / 3,084 (79.3)	3,632 (78.9) / 819 (81.3)	3,494 (79.7) / 749 (81.6)	3,388 (79.9) / 771 (80.9)	3,262 (79.9) / 934 (81.6)	3,010 (80.0) / 859 (80.1)	2,724 (80.6) / 639 (80.1)	2,514 (80.4) / 718 (80.6)
Dad born in Australia, no (%)	3,526 (69.0) / 2,798 (72.0)	3,260 (70.8) / 785 (77.9)	3,156 (72.0) / 728 (79.3)	3,070 (72.4) / 767 (80.5)	2,971 (72.7) / 885 (77.3)	2,765 (73.5) / 827 (77.1)	2,514 (74.4) / 630 (78.9)	2,316 (74.1) / 689 (77.3)
Underweight, no (%)	NA^a^	124 (2.69) / 29 (2.88)	144 (3.28) / 30 (3.27)	126 (2.97) / 29 (3.04)	96 (2.35) / 28 (2.45)	117 (3.11) / 33 (3.08)	105 (3.11) / 23 (2.88)	85 (2.72) / 19 (2.13)
Overweight, no (%)		828 (18.0) / 185 (18.4)	826 (18.8) / 188 (20.5)	607 (14.3) / 121 (12.7)	580 (14.2) / 151 (13.2)	539 (14.3) / 151 (14.1)	510 (15.1) / 101 (12.7)	494 (15.8) / 130 (14.6)
Mild-to-moderate obesity, no (%)		456 (9.90) / 78 (7.74)	414 (9.44) / 71 (7.73)	309 (7.28) / 52 (5.46)	348 (8.52) / 77 (6.72)	322 (8.55) / 69 (6.43)	267 (7.90) / 50 (6.27)	251 (8.03) / 51 (5.72)
Severe obesity, no (%)		178 (3.86) / 32 (3.17)	166 (3.78) / 26 (2.83)	83 (1.96) / 10 (1.05)	50 (1.22) / 9 (0.786)	30 (0.797) / 5 (0.466)	32 (0.946) / 4 (0.501)	45 (1.44) / 8 (0.898)

NA: not applicable. It is not applicable as the definition of these unhealthy weight statuses used in this study cannot be used for wave 1, as in wave 1 BMI percentile was not recorded.

**Table 3:** Number of cases with missing values for independent and dependent variables.

Variable \ Wave	1	2	3	4	5	6	7	8
BMIz and BMI percentile	NA	84	62	50	87	192	212	200
PME^a^ of meat	45	NA						
PME of fish								
PME of dairy								
PME of eggs								
PME of other foods								

PME: prenatal maternal exclusion (from diet).

### Effect of PMDE on BMIz

None of the effects of PMDE variables on child BMIz were statistically significant.

### Effect of PMDE on weight status

Table 4 shows that the PM exclusion (PME) of other foods from the diet, when associated with a different odds ratios of unhealthy weight status, was universally associated with a lower rate of unhealthy weight statuses. The PME of meat was not significantly associated with child weight status. The PME of dairy was associated with a higher rate of underweight in wave 5 and a lower rate of overweight in wave 8. The PME of fish was associated with a higher rate of underweight in wave 8 and mild-to-moderate obesity in wave 4. The PME of egg was associated with a higher risk of overweight during wave 8. Finally, the PME of other foods was associated with a lower risk of overweight in wave 3, mild-to-moderate obesity in wave 5, and underweight, and mild-to-moderate obesity in wave 7.

**Table 4:** Effect of PMDE on weight status odds ratio (95% confidence interval are indicated in parentheses).

Variable\Wave	2	3	4	5	6	7	8
Sample size	1,008	918	953	1,145	1,073	798	891
PME^a^ of meat	NS^b^	NS	NS	NS	NS	NS	NS
PME of fish	NS	NS	MTMO:^c^ 3.424 (1.338, 8.764)	NS	NS	NS	UW:^d^ 4.154 (1.121, 15.39)
PME of dairy	NS	NS	NS	UW:^e^ 5.560 (1.813, 17.05)	NS	NS	OW:^e^ 0.3530 (0.1253, 0.9947)
PME of egg	NS	NS	NS	NS	NS	NS	OW: 5.320 (2.023, 13.99)
PME of other foods	NS	OW: 0.6010 (0.4209, 0.8584)	NS	MTMO: 0.5681 (0.3264, 0.9887)	NS	UW: 0.3527 (0.1246, 0.9988)	NS
						MTMO: 0.4363 (0.2078, 0.9163)	

PME: prenatal maternal exclusion.

NS: nonsignificant.

MTMO: mild-to-moderate obesity.

OW: overweight.

UW: underweight.

### Mediation analysis

None of the mediation effects were statistically significant.

### Moderation analysis

As shown in Table 5, all significant moderation effects on BMIz involved folate supplementation and meat or fish exclusion and lead to higher BMIz. Specifically, the moderation effect of folate supplementation on the effect of PM meat exclusion was associated with increased BMIz in wave 4. The moderation effect of folate supplementation on the effect of PM fish exclusion was associated with increased BMIz in waves 2 and 6.

**Table 5:** Moderation effects of prenatal maternal dietary supplement use on prenatal maternal dietary exclusion (95% confidence intervals are indicated in parentheses).

Moderation effect\Wave	2	3	4	5	6	7	8
Sample size for BMIz analysis	1,008	918	953	1,145	1,073	798	891
PMEM^a^×OTCFe^b^→BMIz	NS^c^	NS	NS	NS	NS	NS	NS
PMEM×Folate^d^→BMIz	NS	NS	0.4710 (0.0080, 0.9341)^e^	NS	NS	NS	NS
PMEM×DietS^f^→BMIz	NS	NS	NS	NS	NS	NS	NS
PMEM×RxFe^g^→BMIz	NS	NS	NS	NS	NS	NS	NS
PMEF^h^×OTCFe→BMIz	NS	NS	NS	NS	NS	NS	NS
PMEF×Folate→BMIz	0.5104 (0.0389, 0.9819)	NS	NS	NS	0.5550 (0.0678, 1.042)	NS	NS
PMEF×DietS→BMIz	NS	NS	NS	NS	NS	NS	NS
PMEF×RxFe→BMIz	NS	NS	NS	NS	NS	NS	NS
PMED×OTCFe→BMIz	NS	NS	NS	NS	NS	NS	NS
PMED×Folate→BMIz	NS	NS	NS	NS	NS	NS	NS
PMED×DietS→BMIz	NS	NS	NS	NS	NS	NS	NS
PMED×RxFe→BMIz	NS	NS	NS	SME^j^	SME	SME	SME
PMEE^i^×OTCFe→BMIz	SME	NS	NS	NS	NS	NS	NS
PMEE×Folate→BMIz	NS	NS	NS	NS	NS	NS	NS
PMEE×DietS→BMIz	NS	NS	NS	NS	NS	NS	NS
PMEE×RxFe→BMIz	NS	NS	NS	SME	NS	SME	SME
PMEO^k^×OTCFe→BMIz	NS	NS	NS	NS	NS	NS	NS
PMEO×Folate→BMIz	NS	NS	NS	NS	NS	NS	NS
PMEO×DietS→BMIz	NS	NS	NS	NS	NS	NS	NS
PMEO×RxFe→BMIz	NS	NS	NS	NS	NS	NS	NS
Sample size for BMIz analysis	979	888	924	1,117	1,040	775	872
PMEM×OTCFe→BWS^l^	NS	SME	NS	NS	4.892 (1.092, 21.91)	NS	SME
PMEM×Folate→BWS	NS	SME	NS	NS	NS	NS	SME
PMEM×DietS→BWS	NS	SME	NS	NS	NS	0.0899 (0.0085, 0.9486)	SME
PMEM×RxFe→BWS	NS	SME	NS	SME	SME	NS	SME
PMEF×OTCFe→BWS	NS	SME	NS	NS	5.509 (1.437, 21.11)	NS	SME
PMEF×Folate→BWS	NS	SME	5.828 (1.201, 28.28)	5.198 (1.291, 20.93)	3.976 (1.004, 15.76)	NS	SME
PMEF×DietS→BWS	NS	SME	NS	NS	NS	NS	SME
PMEF×RxFe→BWS	NS	SME	NS	NS	SME	NS	SME
PMED×OTCFe→BWS	NS	SME	NS	NS	NS	NS	SME
PMED×Folate→BWS	NS	SME	NS	NS	NS	NS	SME
PMED×DietS→BWS	NS	SME	NS	NS	NS	NS	SME
PMED×RxFe→BWS	SME	SME	SME	SME	SME	SME	SME
PMEE×OTCFe→BWS	0.0790 (0.0067, 0.9374)	SME	17.96 (1.685, 191.5)	NS	SME	NS	SME
PMEE×Folate→BWS	NS	SME	17.78 (1.172, 269.8)	NS	SME	SME	SME
PMEE×DietS→BWS	NS	SME	NS	NS	NS	NS	SME
PMEE×RxFe→BWS	SME	SME	SME	SME	SME	SME	SME
PMEO×OTCFe→BWS	NS	SME	NS	NS	NS	NS	SME
PMEO×Folate→BWS	NS	SME	NS	2.716 (1.109, 6.651)	NS	NS	SME
PMEO×DietS→BWS	NS	SME	NS	NS	NS	NS	SME
PMEO×RxFe→BWS	NS	SME	SME	NS	NS	NS	SME

PMEM: prenatal maternal (PM) exclusion of meat from diet.

OTCFe: PM over the counter (OTC) iron supplement use.

NS: nonsignificant.

Folate: OTC folate supplement use.

Moderation effects involving BMIz should be interpreted as how much the BMIz changes with each one unit increase in the moderator (which appears after the multiplication sign) when the independent variable is held constant at 1.

DietS: OTC use of other dietary supplements.

RxFe: prescription use of iron supplements.

PMEF: PM exclusion of fish from diet.

PMEE: PM exclusion of egg from diet.

SME: singular matrix errors.

PMEO: PM exclusion of other foods from diet.

BWS: binary weight status. Moderation effects with BWS as the outcome variable are all given as odds ratios of overweight/obesity relative to the reference category of healthy weight.

Over-the-counter (OTC) iron supplementation had a moderation effect on PM meat exclusion that was associated with an increased risk of overweight or obesity in wave 6. OTC iron supplementation also had a moderation effect on the PME of fish that was associated with an increased risk of overweight or obesity in wave 6. OTC iron supplementation also had a moderation effect on the PME of egg that was associated with a reduced risk of overweight or obesity in wave 2 and an increased risk in wave 4. The moderation effect of folate supplementation on the PME of fish was associated with an increased risk of overweight and obesity in waves 4 to 6. Folate supplementation had a moderation effect on the PME of egg that was associated with an increased risk of overweight and obesity in wave 4. Folate supplementation also had a moderation effect on the PME of other foods that was associated with an increased risk of overweight and obesity in wave 5. Finally, dietary supplementation had a moderation effect on the PME of meat that was associated with a lower risk of overweight and obesity in wave 7.

## Discussion

### Key results

No association was found between PMDE and child BMIz, except with regard to other foods in wave 6.

The PME of meat was not associated with any change in the risk of unhealthy weight statuses such as overweight and obesity at any stage of childhood. When the PME of fish and eggs was associated with a different odds of unhealthy weight statuses, it was associated with higher odds. When PME of other foods was associated with a different risk of unhealthy weight statuses, it was associated with lower odds. The effects of the PME of dairy were more mixed.

No mediation effect on BMIz and weight status was observed. This finding suggested that child birthweight did not play a significant role in mediating the effects of PMDE on child BMIz and overweight/obesity risk. Moderation effects involving PM dietary supplementation were associated with higher BMIz and usually were associated with a higher risk for overweight/obesity, although exceptions exist. Most significant moderation effects involved folate or iron supplementation, although one involving other dietary supplements was observed.

### Limitations

This study had numerous limitations. Among them, data had to be assumed to be missing completely at random, which was apparently false (Table 2). Some ethnic minorities were apparently under-represented in the cleaned data set, and the possibility that dietary exclusion was for medical reasons cannot be controlled for. The effect of dietary exclusion also cannot be controlled at different stages of pregnancy, despite the known differences in the effect of fetal malnutrition at different stages of pregnancy. This finding was based on an observational study, so causal inferences cannot be made according to these results.

### Interpretation

The PME of meat likely has no effect on childhood obesity or overweight risk. The PME of fish may be associated with an increased risk of mild-to-moderate obesity at ages 6 or 7 years and underweight at ages 14 or 15 years. The PME of egg may be associated with an increased risk of overweight at ages 14 or 15 years. The PME of dairy appears to be associated with a more mixed effect on child unhealthy weight status risk, with a higher risk of underweight at age 8 or 9 years and a lower risk of overweight at age 14 or 15 years. The PME of other foods appears to be associated with a lower risk of unhealthy weight status, specifically with a lower risk of overweight at age 4 or 5 years, mild-to-moderate obesity at age 8 or 9 years, and underweight and mild-to-moderate obesity at age 12 or 13 years. PM dietary supplement use had a mixed effect on this relationship and was sometimes associated with higher risks of child unhealthy weight status.

Keeping in mind the data came from an observational study, these results generally indicated that fish and egg were probably food items that were beneficial for expectant mothers to consume. Perhaps this finding was due to eggs and fish having nutrients unique to them that are important for preventing the development of childhood obesity. These nutrients are likely not vitamin B12, choline, iron, or zinc, because other animal products also contain these nutrients. Further studies are needed to identify these nutrients. The exclusion of food items besides dairy, egg, meat, and fish from the mother’s prenatal diet was likely beneficial to the child. Further studies are needed to clarify exactly which food items, when excluded from the mother’s diet during pregnancy, have a beneficial effect on the child’s risk of becoming overweight or obese. There is the possibility that these food items are highly processed and calorically dense, however, it is important to note that this assertion remains speculative until further research is conducted. Dietary supplements did not appear to reduce the risk of unhealthy weight status associated with dietary exclusion. However, this data set recorded only whether these supplements were used during pregnancy, not when, their quantity, frequency of use, nor any underlying conditions they were taken to treat. These observations supported the hypothesis that iron may be an important micronutrient during pregnancy when deciding the child’s later weight status. The observed effect may be due to an underlying deficiency of iron that these supplements were meant to treat.

The human studies most closely identified with this one are those involving dietary supplements or adherence to a Mediterranean diet and their effect on childhood obesity or overweight risk [14, 43]. Adherence to a Mediterranean diet is not correlated with BMIz or childhood obesity or overweight risk but is associated with lower waist circumference, another measure of child adiposity. The Mediterranean diet is rich in fish, olive oil, fruits, vegetables, and unprocessed cereals and is moderate in lean meat and dairy [44]. The current work corroborated the importance of the fish component of the Mediterranean diet. Dietary supplements were found to have no impact on childhood obesity and overweight risk. Thus, the effect of dietary supplements may depend on the mother’s diet. More studies should ideally investigate whether the mother’s nutritional status impacts this effect.

These findings can, if corroborated by further studies that can be better control for possible confounders, lead to changes to the Australian Government’s campaign for the healthy pregnancy that explicitly recommend pregnant women not to abstain from egg and fish during pregnancy. Hopefully, future studies will ascertain exactly what components of these foods are essential for preventing childhood obesity and overweight in the offspring. This information can be used to ensure that women who abstain from these foods for ethical, health, or other reasons still be able to provide their babies with the nutrition they need for the best possible start in life.

### Generalizability

This study was solely conducted within the Australian context. Hence, the results probably cannot be generalized to other populations.
